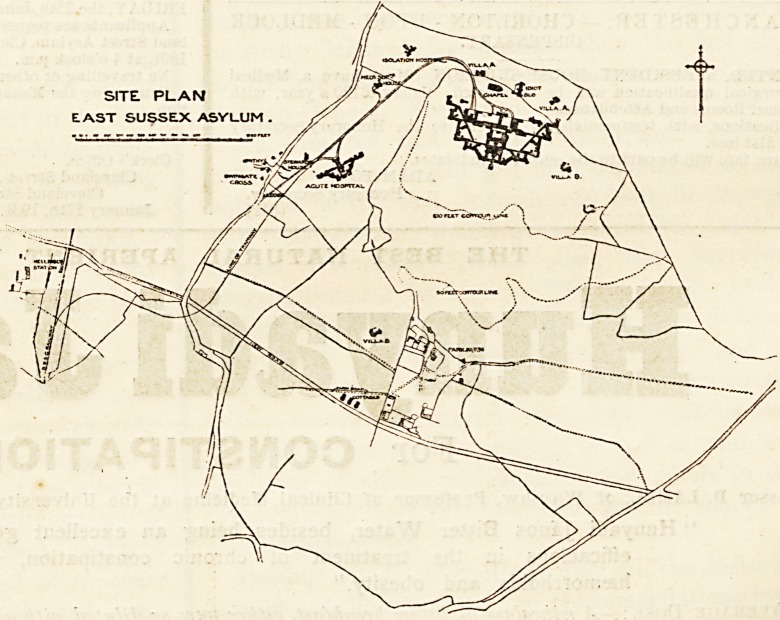# The New Asylum for East Sussex

**Published:** 1901-01-26

**Authors:** 


### THE INSTITUTIONAL WORKSHOP.

### THE NEW ASYLUM FOR EAST SUSSEX.

THIS, the most recently planned of our county asylums, is now being erected near near Hellingly, and it ought to represent the best ideas known regarding the construction of such buildings, more especially as it is evident from the descriptive pamphlet, written by Dr. Newington, that the committee not only went to much trouble and expense in visiting other institutions of a similar kind, but that they had a free hand with the plans. "We claim," says Dr. Newington, "with one or two exceptions, perhaps, no particular novelty in ideas; but we can, I think, claim as novel the arrangement of some ideas already in existence." Probably this is the most that can be said of the plan. In its component parts it is, in fact, a sort of combination of the quadrangular and broad arrow arrangements, or, as Dr. Newington calls it, the "strung bow" plan.

To the critic of to-day one of the most discouraging points met with when studying asylum plans is the conviction that the advance lately made has been extremely little; while in their sister institutions, general hospitals, the advance has been extremely great. More than one cause for this could be named, but these causes need not be referred to at present.

The East Sussex Asylum may be divided into a main building for chronic cases, an annexe for acute cases, and a separate block for idiots. There are also an isolation hospital and villas arranged for 30 patients each. The total accommodation is for 1,115 patients, but it may be extended to 1,275.

Taking the main building first we have, projecting from the centre of the south front, a block for the assistant medical officers. This block is connected to the main building by a corridor, which passing northwards divides the men's hospital ward from the women's; and this juxtaposition enables both hospital wards to be under the same immediate management if this were thought desirable. Lately a scheme similar to this was elaborated by Dr. Steen, of the West Sussex Asylum. The women's hospital ward is designed for 37 beds. These are distributed in two dormitories and in nine single-bedded rooms. One single-bedded room only has a southern exposure. This with its fellow on the men's side and presumably two on the first floor are all the single rooms in the main building which face the south unless we add three in each epileptic block which look either south-east or south-west. The larger dormitory of the hospital ward is for twenty beds. The whole of one side of the ward faces south, and has a sufficient number of windows; but the other side is for about one-third of its length blocked by attendant's room, bathroom, and closet. The latter is the only one provided for the dormitory, and it is not cut off from the dormitory by a ventilating passage. Both ends of the dormitory are blocked—one end by a single room and a passage, and the other by a passage and by part of the smaller dormitory. The latter has eight beds, and more than half of it is embedded in what ought to have been corridor space. Clearly every part of this dormitory ought to have projected from the main line. In its present form cross-ventilation is impossible in part of it, and of course there is a corresponding loss of sunshine. The day-rooms face south-east, and they must be bright and cheerful rooms; but cross-ventilation has hardly been attempted in them. The closets are placed in a block connected by an efficiently cross-ventilated passage.

The next ward is for the sick and infirm. It is intended for 48 patients. There are two dormitories, one of which is bent on itself at an obtuse angle. It has 19 beds, and efficient cross-ventilation is provided for about two-thirds of these. The other dormitory is also for 19 beds. The side facing east is almost entirely free, but the west side is partly blocked by a bathroom and a closet; and again the latter is innocent of any cross-ventilated passage. Both ends of this dormitory are entirely blocked by partition walls, each of which, however, has a door, which might, perhaps, be used for end ventilation. The east side of the dormitory has five windows, and the west four, so that cross-ventilation may be fairly well carried out. One of the single-bedded rooms is placed outside the corridor and faces east; the others look north and north-east into an enclosed court where the sanitary block is placed.

There are two day-rooms. In one cross-ventilation has been obtained and in the other not. The block for baths, lavatories, and closets is well arranged and properly cut off from the main. Eastward is the acute wards for 40 patients. One dormitory contains eight beds. It has the same fault as that spoken of in the hospital ward. Half of it is driven inwards towards the corridor instead of having three of its sides entirely exposed. The other dormitory contains 19 beds; and although both ends are again blocked it may be said to have the best means of cross-ventilation of any dormitory in the building. One closet near the smaller dormitory has no cross-ventilating passage, but the sanitary block attached to the day-rooms is correctly designed. There are three day-rooms, all pleasant-looking rooms, and two possess diagonal ventilation. In fact, having regard to the glazed partitions, some of which will, no doubt, be made to open, it might be said that cross-ventilation is here obtained. Although none of the day-rooms are perfect, the author of the plans, speaking generally, has been more successful with them than with the dormitories.

The Epileptic Ward comes next. It is for 51 patients, and is provided with two day-rooms which adjoin each other. One of these rooms has two sides free, but the other is blocked on three sides; but there are large bay windows in each ward. Indeed, the profusion of bays is one of the most striking and pleasing features of the whole plan. The dormitory contains 41 beds, which are placed in four rows. Thus the room must be about 37 feet wide, and it is much to be feared that, in calm weather at any rate, thorough perflation of the air will not be obtained. There are, however, five windows on each side of the dormitory. The remaining block is at the north-east corner. It contains 110 chronic patients, and is three storeys in height. We are of opinion that no part of an asylum should exceed two storeys, and that patients should sleep on the same floor they have occupied by day; but apart from these structures, and as far as can be judged from the ground plan, the block is well adapted for its purpose. Reviewing the whole plan, one of our gravest objections is to the absence of ward dining-rooms. Either the patients must be taken three times a day to the recreation hall for their meals or they must dine in their day-rooms, and it is hard to say which alternative is the worst.

The Administration Block may be taken as a very fair sample of the present favourite form of arrangement, but there is nothing in it calling for special attention.

The Acute Hospital is planned for 32 male and 48 female patients; and "it is to be devoted solely to recoverable patients." The hospital is also on the "strung bow" principle, and each wing is described by Dr. Newington as "divided into three sections, the outer being for the more excited cases, the middle for the quieter and more depressed types, while inside will be found the convalescent."

### EAST SUSSEX ASYLUM.

GROUND PLAN

| REFERENCE TABLE | |
|---|---|
| S. SINGLE ROOM | B. BATH |
| Bᵀᴿ BOOT ROOM | Sᴸ SLOPS |
| L. LAVATORY | D.L. DIRTY LINEN |
| W.S WARD SCULLERY | C. COALS |
| ST STORE | Sˢ SCULLERY |
| A. ATTENDANT | W. WATER CLOSET |

| ACCOMMODATION | MALES | FEMALE | TOTAL |
|---|---|---|---|
| HOSPITAL WARDS | 60 | 74 | 134 |
| SICK AND INFIRM | 70 | 96 | 166 |
| RECENT AND ACUTE | 80 | 80 | 160 |
| EPILEPTICS | 80 | 100 | 180 |
| CHRONICS | 90 | 110 | 200 |
| TOTAL MAIN ASYLUM | 380 | 460 | 840 |
| ACUTE HOSPITAL | 30 | 50 | 80 |
| 4 VILLAS | 60 | 60 | 120 |
| IDIOT BLOCK | | | 60 |
| | | TOTAL | 1100 |

"The dormitories are above, and the day-rooms on the ground floor." How this principle will work out in practice remains to be seen; but few medical superintendents would like the prospect of removing eighty acute cases from the ground floor every night and bringing them back every morning. Such transferring cannot be effected without much trouble, and also, it is to be feared, without considerable risk, especially as no "lifts" are provided. An exercising gallery is an innovation—at least we do not remember having met with it before—but we can readily believe it to be a useful addition to an asylum. There are four sanitary spurs containing the closets properly separated from the main building by ventilating passages; but in five other cases no such passages exist—strange omissions from a twentieth-century asylum. In each wing one day-room has two of its sides free; but in other instances half at least of the day-room space is embedded. In our remarks on the main building we said that the day-rooms were better than the dormitories. Here, on the whole, the opposite would hold good. It should be stated that many of the single-bedded rooms have a southern exposure. The acute hospital is placed about 280 yards from the main building and faces south-east. It is not possible to determine what advantage accrues from its being a detached building. If it were split into two wings, and each wing connected with the main building by a corridor, it would be equally useful as a curative agent and vastly more convenient; so at least it strikes us; but if the principle be found successful in practice, theoretical objections are of no great value. No separate block is provided for private patients. This acute block with some modifications would be suitable for this purpose, and it would not surprise us much to find it one day used for it. There is a block for 60 idiot children, "which follows with certain variations the general ideas of the excellent house at Fareham." There are four detached villas, and these seem well arranged for their respective purposes, namely, to accommodate such patients as work in the laundry and at needlework, or are employed in the shops and on the farm.

One of the best features of the asylum is that the medical superintendent's house is entirely detached; but it is a matter to be much regretted that the chapel has not been incorporated with the main building, which could easily have been done with great advantage to patients and staff.

The architect seems to have been appointed and the plans adopted without any public or even limited competition. To this private system we believe the greatest objections attach themselves. It strikes at the very root of that wholesome rivalry from which improvement is to be looked for; and we hope that no County Council will ever again be induced to follow such a system unless all the attending circumstances are exceptional. It has been said in these columns that an asylum should be built for £150 a bed Since that was said building prices have greatly advanced; but we still believe that an asylum in every way efficient could be built for less than £200 a bed. Dr. Newington says the East Sussex Asylum is to cost £275 a bed. Why this extravagance in an institution for rate-supported patients?

## Figures and Tables

**Figure f1:**
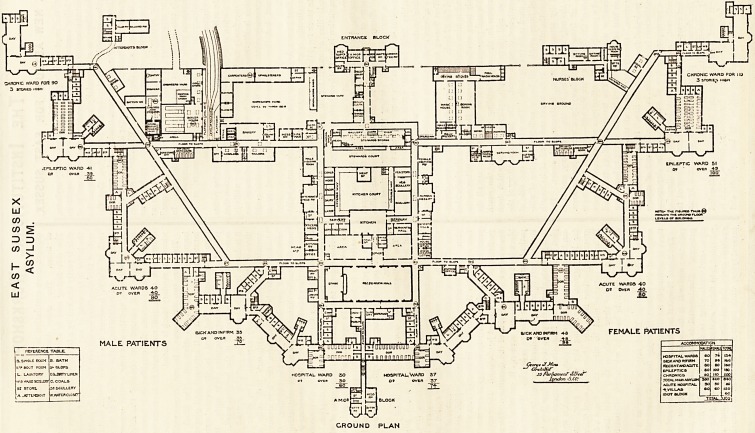


**Figure f2:**
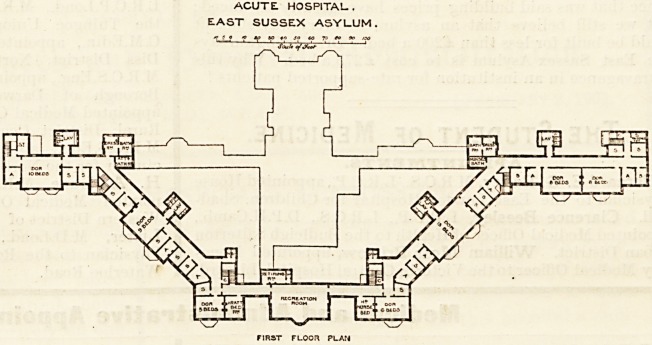


**Figure f3:**
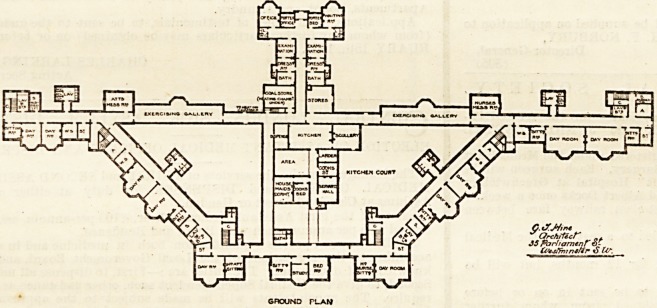


**Figure f4:**